# Autologous Blood Patching to Mitigate Persistent Air Leaks Following Pulmonary Resection: A Novel Approach

**DOI:** 10.7759/cureus.7742

**Published:** 2020-04-20

**Authors:** Kenneth Dye, Samuel Jacob, Mojahid Ali, David Orlando, Mathew Thomas

**Affiliations:** 1 Cardiothoracic Surgery, Mayo Clinic, Jacksonville, USA; 2 Surgery, Mayo Clinic, Jacksonville, USA

**Keywords:** autologous blood patching, air leak, pulmonary resection, emphysema, mass resection

## Abstract

Background

Autologous blood patch (ABP) utilized as a visceral pleural sealant for air leak post lung resection has been well documented in medical literature.

Purpose

To present our experience of a novel approach, we employed to instill autologous blood into the pleural space to mitigate persistent air leaks following pulmonary resection.

Methods

From January 2007 to September 2011, 19 patients were submitted to autologous blood patching for persistent air leaks following surgery. Demographic and surgical characteristics were collected at baseline. Blood patching measures were recorded at the time of the event. Continuous variables were summarized with median and range while categorical measures were summarized with frequency and percent. Due to the small sample size and descriptive nature of this study, no hypothesis tests were performed. All analyses were conducted using R Statistical Software.

Results

The median age of patients who required a blood patch for a persistent air leak was 67.9 (Range: 50.3-78.7) years and 11 (57.9%) were males and 8 (42.1%) were females. The majority (78.9%) of the patients’ first surgery was mass resection and 4 (21.1%) had a lung volume reduction. Seven (36.7%) required a re-do surgery, and almost all (89.5%) had 28 mm chest tubes used during surgery. The majority or 63.2% (N=12) of the patient's air leaks were classified as moderate, 21.1% (N=4) as severe, 15.8% as mild (N=3); twelve (63.2%) required one attempt for a successful blood patch, 6 (31.6%) required two attempts, and one (5.3%) required three which were all unsuccessful. The median number of days from detecting air leaks to blood patch for the air leak that required two attempts was 9 (Range: 8, 23) days for lung volume reduction patients and 16 (Range: 6, 26 ) days for mass resection patients.

Conclusion

Blood patching remains an effective bedside strategy that can be carried out with minimal risk. We believe opportunities exist to further advance the method of delivering blood as an autologous sealant to mitigate persistent air leaks (PAL).

## Introduction

Persistent air leaks (PAL) following pulmonary resection is common and remains the Achilles’ heel of the thoracic surgeon. PAL has been proven to impact length of stay, contamination of the pleural space, consideration for re-operative surgery, and patient satisfaction [1]. PAL greater than 7 days in duration has been described to occur in up to 26% of patients undergoing pulmonary resection [2]. Various methods have been employed to reduce the frequency and severity of PAL that include aerosolized biologic adhesives and autologous fibrin-based products principally conceived to improve hemostasis during anastomotic surgery. The clinical indications for these agents over time have increased the armamentarium offered to the thoracic surgeon to control PAL following pulmonary resection.

Autologous blood patch (ABP) has been used clinically for nearly 60 years, first described by J.B Gormley, MD, in the Anesthesia literature for management of post-spinal headache following lumbar puncture [3]. Since that time, ABP has been well described in the thoracic literature for management of pleural air leaks for both pneumothorax and iatrogenic induced air leaks following pulmonary resection. To date, methods to instill autologous blood or cryoprecipitate into the pleural space are admitted directly through the terminal end of an indwelling drain by manual methods from a syringe. This method, though effective, results in a large quantity or bolus of autologous blood residing stagnant within the body of the drain. In this manner, the drain is at risk of thrombotic occlusion with reduced blood draining from the pleural cavity. The material and methods will disclose our novel approach to improve the delivery of autologous blood into the pleural space and its impact on our patient population.

## Materials and methods

Nineteen subjects were observed to have ABP for persistent air leaks following surgery between January 2007 and September 2011. All patients were over the age of 18, and one patient was excluded due to inconsistent data in his electronic medical record. Demographic and surgical characteristics were collected at baseline. Blood patching measures were recorded at the time of the event. Continuous variables were summarized with median and range while categorical measures were summarized with frequency and percent. Summary of baseline characteristics are found in Table 1.

**Table 1:** Summary of baseline characteristics

Number of patients	Overall (N=19)
Age at surgery (years)	67.9 (50.3, 78.7)
Sex	
Male	11 (57.9%)
Female	8 (42.1%)
COPD	5 (26.3%)
Emphysema	5 (26.3%)
Type of first surgery	
Lung volume reduction	4 (21.1%)
Mass resection	15 (78.9%)
Re-do surgery	7 (36.8%)
Size of chest tubes	
14	1 (5.3%)
28	17 (89.5%)
Unknown	1 (5.3%)
Severity of Air Leak	
Mild	3 (15.8%)
Moderate	12 (63.2%)
Severe	4 (21.1%)
Variables are summarized as median (minimum, maximum) or N (%).	

Due to the small sample size and descriptive nature of this study, no hypothesis tests were performed. All analyses were conducted using R Statistical Software (version 3.6.1; R Foundation for Statistical Computing, Vienna, Austria).

Blood patch approach:

A bedside “setup” was created with a bundling of supplies such as sterile intravenous connection tubing, towels, scissor, and a sterile clamp. A large bore 2 lumen PICC line was placed prior to proceeding. Several patients early on had direct venipuncture from the antecubital fossae; however, this method was rapidly abandoned due to patient discomfort. On the back table, a length of sterile IV tubing with a side port leur connector was identified. The length of tubing distal to the connector was measured to a length of 13cm beyond the length of a standard chest tube (CT) 15cm. The stopcock connector was placed in the IV tubing circuit and the cut remnant of tubing proximal was clamped to prevent an efflux of blood bi-directionally. Blood was aspirated from the PICC line, the CT disconnected in a sterile manner, and the IV tubing was placed through the CT beyond the body of the drain and the blood administered. We would then flush the tubing and repeat the process until we instilled enough quantity of blood within the pleural space for blood patching. In this manner, we were able to keep the CT clear and minimize obstruction of the tube from stagnant blood within the body of the drain.

## Results

The median age of patients who required a blood patch for a persistent air leak was 67.9 (Range: 50.3-78.7) years and 11 (57.9%) were males and 8 (42.1 %) were females. The majority (78.9%) of the patient's first surgery was mass resection and 4 (21.1%) had a lung volume reduction, (57.9% ) as a lobectomy. Seven (36.7%) required a re-do surgery, and almost all (89.5%) had 28 mm CTs placed. The majority or 63.2% (N=12) of the patient's air leaks were classified as moderate, 21.1% (N=4) as severe, and (15.8% ) as mild (N=3). Twelve (63.2%) required one attempt for a successful blood patch, 6 (31.6%) required two attempts, and one (5.3%) required three which were all unsuccessful (Table 2).

**Table 2:** Patching characteristics and complications by surgery type Variables are summarized as median (minimum, maximum) or N (%).

	Mass resection (N=15)	Lung volume reduction (N=4)	Total (N=19)
Severity of Air Leak			
Mild	2 (13.3%)	1 (25.0%)	3 (15.8%)
Moderate	9 (60.0%)	3 (75.0%)	12 (63.2%)
Severe	4 (26.7%)	0 (0.0%)	4 (21.1%)
Number of blood patches			
1	11 (73.3%)	1 (25.0%)	12 (63.2%)
2	3 (20.0%)	3 (75.0%)	6 (31.6%)
3	1 (6.7%)	0 (0.0%)	1 (5.3%)
Days from air leak to first blood patch	7 (5, 19)	6 (4, 14)	7 (4, 19)
Days from air leak to second blood patch	9 (8, 23)	16 (6, 26)	9 (6, 26)
Volume of blood at first patch (ml)			
45	1 (7.1%)	0 (0.0%)	1 (5.6%)
60	1 (7.1%)	0 (0.0%)	1 (5.6%)
75	0 (0.0%)	1 (25.0%)	1 (5.6%)
80	1 (7.1%)	0 (0.0%)	1 (5.6%)
90	1 (7.1%)	0 (0.0%)	1 (5.6%)
100	3 (21.4%)	1 (25.0%)	4 (22.2%)
120	7 (50.0%)	2 (50.0%)	9 (50.0%)
Volume of blood at second patch (ml)			
80	0 (0.0%)	1 (33.3%)	1 (16.7%)
100	1 (33.3%)	1 (33.3%)	2 (33.3%)
110	1 (33.3%)	1 (33.3%)	2 (33.3%)
140	1 (33.3%)	0 (0.0%)	1 (16.7%)
Hours of leak post blood patch	1 (1, 14)	2 (1, 9)	1 (1, 14)
Number of unsuccessful blood patches			
0	11 (73.3%)	1 (25.0%)	12 (63.2%)
1	3 (20.0%)	2 (50.0%)	5 (26.3%)
2	1 (6.7%)	1 (25.0%)	2 (10.5%)
Complications			
None	8 (53.3%)	2 (50.0%)	10 (52.6%)
Empyema	4 (26.7%)	0 (0.0%)	4 (21.1%)
Other	3 (20.0%)	2 (50.0%)	5 (26.3%)

The median number of days from the discovery of the air leak to the first attempted blood patch was 7 days (Range: 4,19). At least six of the patients that received a lung volume reduction or mass resection required two blood patching attempts, of which five were successful and one was not. The median number of days from air leak to blood patch for an air leak that required two attempts was 9 (Range: 8, 23) days for lung volume reduction patients and 16 (Range: 6,26 ) days for mass resection patients. The volume of blood used to patch air leaks in lung volume reduction patients ranged between between 75 ml to 120 ml on the first attempt and 80 to 110 ml on the second attempt. The volume of blood used for patients with a mass resection varied from 45 ml to 120 ml on the first patch and 140 ml on the second try.

Ten (52.6%) patients did not experience any complications after their blood patching procedure. However, four (21.1%) developed subcutaneous emphysema where two had moderate air leaks and two had severe air leaks. Three of those patients underwent lobectomies and one was a mass resection. No lung volume reduction patients developed empyema, however one patient from the mass resection group developed a pleural space infection (empyema) . Other complications (such as AFIB, lung transplantation, and persistent tachycardia) occurred in five patients where four air leaks were moderate and one was severe. Further stratification revealed that three were lobectomies and two were lung volume reduction surgeries.

## Discussion

Our experience supports blood patching as an efficacious and under-utilized means to seal a breach of the pleural surface following pulmonary resection. ABP, though under-utilized, offers the clinician a safe and effective bedside rescue strategy to manage pleural air leaks that do not seal following prolonged observation. The primary benefit observed by this technique is to obviate the need for re-operative surgery. Despite the recognized advantages and overall low complication rates reported, this procedure it is not without inherent risk. 

Empyema has been an obvious concern by practitioners applying ABP and has been tracked as a consistent metric by most manuscripts reviewed. Intuitively, there remains a hesitancy by most thoracic surgeons to instill blood from a peripheral site into a pleural cavity due to its inherent properties as a culture medium despite the paucity of evidence that this occurs with regularity following ABP. The incidence of space contamination reported by Cagirici et al. was only noted at 9.4% of a 32 patient cohort with prior authors reporting no incidence of empyema or contamination of the pleural space [4]. In our studied patient, we had one case (0.6 %) with a pleural space infection. Williams et al. however, published a case report of near catastrophic tension pneumothorax following direct instillation of phlebotomized blood into a small caliber intercostal drain in a young patient with cystic fibrosis following a spontaneous pneumothorax [5]. Williams et al. additionally discussed the role of drain caliber, length of time of venesection, and the importance of flushing of the drain to reduce the risk of drain thrombosis and lessen this risk. Obstruction in the setting of a large volume air leak appeared to have been the inciting event proposed by Williams et al. as the causative factors, involving hemodynamic instability and a tension pneumothorax. Rapid improvement was noted once drain patency was established by antegrade flushing of the drain.

A unified consensus on drain diameter following pulmonary resection remains a topic of debate. The trauma literature however, does support the use of large caliber drains when faced with high volume viscous drainage such as a hemothorax [6]. Diameter alone does not appear as relevant when dealing with a pneumothorax or persistent air leak alone, where a small caliber drain appear sufficient [7,8]. The recognition that a #28fr Argyle CT possesses an internal volume of 20cc and a #32fr being 25cc is of vital importance, as most clinicians seem to favor the use of 50ml as a starting point or 1ml/kg for injecting blood directly into the body of the drain or CT. A broad review of the literature disclosed that this appears to be an overwhelming method used and a benchmark for quantity. If one does not back flush the chest drain following ABP, 40-50% of the instilled blood will remain stagnant within the body of the drain, potentially rending the tube non-functional, secondary to obstructive internal thrombus. 

We employed a novel method to introduce blood directly inside the pleural space bypassing the CT as the primary conduit of delivery. In this series, we recognized that the slow venesection noted by Williams et al. was important as it translated into visible platelet aggregation likely from catheter size and the negative pressure to obtain blood during phlebotomy [9]. Our observation was that large aliquots of blood during aspiration translated to an excessive push force to dispense blood secondary to high viscosity and visible separation of blood components. We believe the quality of blood used is as important as the amount administered, within reason. The amount of blood obtained per patient to patient was somewhat variable in our hands; however, we did attempt to administer at least 50-60 to 150 cc.

Limitations

This study has limitations, due to the small sample size and descriptive nature of this study. No hypothesis tests were performed. In addition, this is a retrospective study with no control group to compare the results and calculate a "p" value.

## Conclusions

This method has permitted us to direct blood in a more precise manner into the pleural cavity without compromising the patency and integrity of the drain. Blood patching remains an effective bedside strategy that can be carried out with minimal risk. We believe opportunities exist to further advance the method of delivering blood as an autologous sealant to mitigate PAL.
